# Cationic antimicrobial peptide, magainin down-regulates secretion of pro-inflammatory cytokines by early placental cytotrophoblasts

**DOI:** 10.1186/s12958-015-0119-8

**Published:** 2015-11-06

**Authors:** G. Anupa, M. A. Bhat, A. K. Srivastava, J. B. Sharma, N. Mehta, Asmita Patil, Jayasree Sengupta, D. Ghosh

**Affiliations:** Department of Physiology, All India Institute of Medical Sciences, New Delhi, India; Department of Obstetrics and Gynaecology, All India Institute of Medical Sciences, New Delhi, India

**Keywords:** Cationic antimicrobial peptide, Cytokines, Cytotrophoblasts, Early placenta, Inflammatory response, Magainin

## Abstract

**Background:**

Human placental villous cytotrophoblasts exhibit relative externalization of negatively charged moieties to the outer leaflet of the plasma membrane during the time of syncytialization rendering their reactivity to positively charged cationic antimicrobial peptides (CAMPs) during the window of implantation and early placentation. Vaginal administration of a synthetic CAMP, Ala^8,13,18^-magainin II amide (AMA) inhibited blastocyst implantation and early placentation in monkeys. Furthermore, the administration of AMA resulted in significant inhibition of cell differentiation, enhancement in apoptosis and loss of viability in first trimester placental villous cytotrophoblasts in primary culture. The present study examines the effect of in vitro application of different doses (0, 1, 10, 100, 1000 ng/ml) of AMA on the secreted cytokine profiles of cytotrophoblasts obtained from placental villi samples (*n = 13*) collected during 8–9 weeks of gestation and grown on three-dimensional collagen matrix in vitro.

**Methods:**

A panel of forty-eight (48) cytokines in conditioned medium was analysed using multiplex immunoassays technique. Further, the steady state transcript levels of four cytokines (CCL4, CCL5, IL1B, IL6), the concentrations of which were affected by AMA in the isolated cytotrophoblasts, as well as, two cytokines (IL1A and TNF) which were not affected by AMA were estimated. Input list of cytokines secreted by cytotrophoblasts and showing differential secretion in response to AMA were used in enrichment analysis for the generation of biological networks.

**Results:**

Placental cytotrophoblasts secreted 27 cytokines, 13 of which are affected by AMA in vitro with significantly decreased secretion of CCLs-2, 3, 4, 5, CXCLs-1 and 8, FGF2 and MCSF and that of IL1B, IL6 and MIF, and increased secretion of IL16 and IL-2RA. Of the above cytokines showing differential secretion, only IL-2RA, IL16 and MIF showed significant correspondence in the steady state expression of their respective transcript levels. Post-hoc Enrichment analysis revealed Toll-like receptor (TLR) mediated pathways were the top-scored target pathways that were affected by AMA.

**Conclusions:**

Administration of a CAMP causes shift in the balance of immune-inflammatory responses involving downstream pathways of TLRs in cytotrophoblast function. Further verification of functions of placental trophoblasts on administration of CAMP with pregnancy outcome is necessary.

**Electronic supplementary material:**

The online version of this article (doi:10.1186/s12958-015-0119-8) contains supplementary material, which is available to authorized users.

## Background

Cationic antimicrobial peptides (CAMPs) are gene-encoded short chain peptides (10–50 amino acids) having an overall positive charge due to the presence of positively charged amino acids namely lysine, arginine and histidine. CAMPs contribute to body’s innate immune response and are evolutionarily conserved. These peptides are broad-spectrum antibiotics as they impart effective antagonistic action on gram-positive and gram-negative bacteria, protozoa and fungi [1]. Positively charged CAMPs interact with negatively charged bacterial cell membrane by electrostatic force of attraction, which is followed by pore formation in the membrane and irreversible membrane disruption followed by cell death [2, 3]. On the other hand, CAMPs normally show lower affinity towards the mammalian cell membrane because of differences in its composition, characterized by the presence of quantitatively lesser aggregation of negatively charged phospholipids in the outer cell membrane leaflet, and relative abundance of cholesterol as compared to that of bacterial cell membrane [4]. Additionally, transmembrane potential of bacterial cell membrane is more polarized to net negativity as compared to the mammalian cell membrane [5]. However, human placental villous cytotrophoblasts exhibit relative externalization of negatively charged moieties to the outer leaflet of the plasma membrane during the time of syncytialization, thus rendering themselves to be reactive to the positive charged CAMPs during the window of implantation [6]. Thus, there is a need for understanding the action of CAMPs in the process of placentation, as CAMPs appear to be promising in meeting the need for new antibiotics.

Ala^8,13,18^-magainin II amide (AMA) is a synthetic alpha-helical CAMP and it is modified from natural Magainin II obtained from African frog, *Xenopus leavis*. Magainin II bears significantly high anti-microbial activity [7]. Furthermore, replacement of three amino acid residues, namely one serine at position 8 and two glycine residues at positions 13 and 18, respectively with alanine renders an enhanced antimicrobial activity in AMA [8]. Additionally, amidation of its terminal amino acid makes its alpha-helical conformation stabilized and lowers its susceptibility to exopeptidase action [8]. It shows chemical properties that are very similar to that of human alpha-helical CAMP, cathelicidin peptide LL-37 [1]. We have earlier demonstrated using AMA that CAMPs may exert deleterious action on placental cytotrophoblasts during blastocyst implantation and early placentation [9–12], however, the underlying process is not known. In the present study, we proposed to examine the effect of application of a synthetic CAMP on the secreted cytokine profiles of human early placental villous cytotrophoblasts in vitro. In order to fulfill the projected objective of the study, isolated villous cytotrophoblasts from human placental villi collected during 8–9 weeks of gestation were maintained in primary culture and exposed to different doses of AMA to simultaneously examine a panel of forty-eight (48) cytokines in conditioned medium using multiplex immunoassays technique [13, 14]. Finally, we have examined the steady state transcript levels of the candidate cyto/chemokines, the concentrations of which were affected by AMA to examine the probable level of action of AMA on the isolated cytotrophoblasts.

## Methods

### Tissue samples and chemicals

Human placental samples (*n* = 13) were obtained from women (age: 22–35 years) undergoing elective surgical termination of singleton pregnancy between 8 and 9 weeks of gestation (timed from last menstrual period) without undergoing any prior medication. All women provided their written informed consents. The Ethics Committee of the All India Institute of Medical Sciences approved the research study. Placental samples were collected in sterile ice-cold phosphate buffered saline (PBS, pH 7.4) and transported on ice to the laboratory within 10 min after collection for further processing. The chemicals were obtained from Sigma Chemical Co. (St. Louis, MO, USA), if otherwise is not stated.

### Isolation of placental villous trophoblast cells

Cytotrophoblasts were isolated from freshly collected placental villi as described previously [11, 12, 15]. Briefly, villous tissues (~3 g) were dissected from chorionic membranes and washed with sterile cold Ca^2+^/Mg^2+^ free PBS (pH 7.4) containing gentamycin (50 μg/ml) and D-glucose (1 mg/ml). Placental tissues were incubated in an enzyme mixture [0.25 % (w/v) trypsin, 0.02 % (w/v) deoxyribonuclease type-I (DNase I), 15 mM HEPES, 5 mM magnesium sulphate, penicillin (100 IU/ml), streptomycin (100 μg/ml) and amphotericin B (2.5 μg/ml)] at 37 °C initially for 30 min and then for another three cycles of 10 min each. Cell suspension was passed through a pre-equilibrated mesh filter (pore size 60 μm) to remove cellular debris. The filtrate was subjected to enrichment on a preformed 10 to 70 % Percoll gradient at 800× g for 20 min at 20 °C. The mononuclear cells were immunopurified by depletion of CD45-positive leucocytes using MACS microbeads conjugated with monoclonal mouse antibody against CD45 and magnetic separation columns type LS in combination with a MidiMACS separator (Miltenyi Biotec, Bergisch Gladbach, Germany) [11, 12]. The negative fraction containing an enriched villous trophoblast cell population was collected and immunocharacterised for cytokeratin 7 (CK-7), βhCG, vitronectin receptor (CD51), vimentin (Vim) and von Willebrand factor (vWF) as described below.

### Cell culture

The methodological details of cytotrophoblast culture have been detailed previously [12]. Briefly, isolated mononucleated cytotrophoblast cells were plated at a density of 1 × 10^5^/cm^2^ on rat-tail collagen I and cultured at 37 °C in a humidified air atmosphere of 5 % CO_2_ and in complete medium [DMEM: F12 (1:1), 10 % (v/v) fetal calf serum, penicillin (100 IU/ml), streptomycin (100 μg/ml), amphotericin B (2.5 μg/ml)] for 24 h to allow for their attachment to collagen. Subsequently, the cells were maintained in serum-free medium with antibiotics and antimycotics as described above and supplemented with insulin (5 μg/ml), transferrin (5 μg/ml), selenium (5 ng/ml) and hydrocortisone (0.5 μg/ml). The same pools of cells were treated in triplicates without or with AMA at different doses (1, 10, 100 and 1000 ng/ml) based on our previous observation that AMA at these concentrations affected synthetic capacity for hCG and hPL without affecting the viability of the target cells at 24 h in primary culture [11, 12]. Cells were harvested at 24 h to obtain cells for immunocytochemical characterisation of cells and RNA extraction, and conditioned medium for immunoassays and immunoblot experiments as described below.

### Multiplex assays of cytokines in conditioned media

The details of 48 cytokines, chemokines and growth factors have been reported elsewhere [13, 14] and are shown in Additional file 1: Table S1. Cell culture supernatants were assessed by quantitative cytokine assays using a Bioplex™ Pro-human cytokine 27-plex panel and a cytokine 21-plex panel based on xMAP technology (Bio-Rad Laboratory, Hercules, CA, USA) according to the pre-optimised protocol as described earlier [13, 14]. Briefly, antibody–conjugated beads were added to individual wells of a 96-well filter plate and adhered using vacuum filtration. After washing, 50 μl of prediluted standards or conditioned media were added, and the filter plate was shaken at 300 rpm for 30 min at room temperature. A prediluted multiplex biotin-conjugated detection antibody was then added for 30 min. Prediluted streptavidin-conjugated phycoerythrin was added followed by an additional wash and the addition of Bio-Plex assay buffer. The filter plate was analysed, and concentrations of each cytokine were determined using the Bio-Rad Bio-Plex 200 instrument equipped with BioManager v6.0 software (Bio-Rad). All samples in triplicates were assayed in a single run. Standard curves were generated for each biomarker. Goodness of fit for standard curves was determined by the standard recovery method and by calculating the concentration of each standard [16]. The intra-assay variations were less than 10 %. Only those cytokines were selected that could be detected at ≥0.1 pg/mg Bradford protein in at least 80 % of cultures.

### Immunocytochemistry

After termination of culture at 24 h, cells treated with and without AMA (1000 ng/ml) were subjected to immunofluorescent staining using antibodies against cytokeratin 7 (CK-7), βhCG, vitronectin receptor (CD51), vimentin (Vim) and vWF (see Additional file 2: Table S2 for details of the antibodies used) to check the purity of the cells using samples (*n* = 5) in duplicates for both treatment groups as described earlier [12]. It was assumed that the purity of cells treated with other concentrations (1, 10 and 100 ng/ml) of AMA would be similar. Appropriate fluorochrome-conjugated secondary antibodies (Molecular Probes, Grand Island, NY, USA) were used for visualization. Specificity of the antibody binding was assessed by omitting primary antibodies, immunoadsorption of primary antibodies with target antigens, replacing primary antibodies with unrelated IgG from same species and other species, omitting secondary antibodies, and replacing secondary antibodies with unrelated IgGs from same or other species in parallel cultures.

### Western blotting

Based on the *post-hoc* analysis of the immunoassays as described below, two (2) proteins - CCL4 (MIP1B) and TNF - were chosen to assess their expressions in conditioned medium by employing Western immunoblot technique as secretion of CCL4 was affected, while TNF was both very low in medium and was not affected in isolated cytotrophoblasts following application of AMA in vitro. The methodological details have been described elsewhere [12, 13]. After termination, conditioned medium obtained from cultures with cells treated without (Control, +cells) and with 1000 ng/ml AMA (AMA, +cells), as well as, unconditioned medium containing 1000 ng/ml AMA (Control, −cells) from different sets (*n* = 3) of experiments were subjected to Western immunoblot using antibodies against CCL4 and TNF (see Additional file 2: Table S2 for details of the antibodies) as described earlier [12, 13]. Briefly, 20 μg Bradford protein for each sample and the pre-stained molecular weight markers were separated by SDS-PAGE and subsequent Western immunoblotting techniques were done using nitrocellulose membranes and chemicals obtained from Bio-Rad (Hercules, CA, USA). Final visualisation was achieved using Vectastain ABC immunoperoxidase kits (Vector Laboratories, Burlingame, CA, USA). Respective primary and secondary antibody controls were run simultaneously to examine the specificity of the antibodies. The molecular weights and semi-quantitative analysis of the bands were determined using densitometric equipment (Pharos FX Plus Molecular Imager) and optimised densitometric analysis software (Quantity One) from Bio-Rad (Hercules, CA, USA). The measures of optical densities were performed from the log of inverse of transmittance for each target antigen and that of total stained bands as total secreted protein from 20 μg Bradford protein. The measures of optical densities for individual immunopositive antigens were calculated as per cent of total integrated optical densities [17].

### Quantitative real time RT-PCR

The relative expression of nine (9) genes of reported cytokines, four (CCL4, CCL5, IL1B, IL6) of which showed reduced, two (IL1A and TNF) of which did not show any change and two (IL-2RA and IL16) of which showed increased secretion following application of AMA to the cytotrophoblasts were selected as targets from the *post-hoc* analysis (as described below) of the Bio-Plex cytokine data. All samples were assessed using SYBR Green-based quantitative RT-PCR protocol according to MIQE guidelines using two most consistently expressed house-keeping genes, namely glyceraldehyde 3-phosphate dehydrogenase (GAPDH) and ubiquitin C (UBC) as endogenous controls. The cells treated without and with 1000 ng/ml AMA (*n* = 5/each) yielding consistent secretory profile were employed in this experiment as most significant results were obtained between these two groups. Thus, the chosen five samples were a subset of original thirteen samples. The methodological details were given elsewhere [18]. Briefly, total RNA was extracted using Trizol (Agilent Technologies Singapore Pvt. Ltd., Shung Avenue, Singapore), purified with DNase I and subjected to re-extraction when necessary. The yield and purity of the extracted RNA were verified using standard spectrophotometric methods and 1 % agarose gel electrophoresis. Furthermore, the RIN score of individual samples was determined using the Agilent 2100 Bioanalyzer, RNA 6000 NanoLabChip kit and Agilent 2100 Expert Software (Agilent Technologies, Inc., Santa Clara, CA, USA). The samples yielded sufficient amounts of RNA and an acceptable RIN score (>8.0) and those RNA samples were used for the experiment. For the real time RT-PCR, the first-strand cDNA was synthesised from 2 μg of total RNA with an optimised RevertAid™ First Strand cDNA Synthesis Kit (Fermentas, Germany), and the PCR was performed using SYBR Green/Fluorescein qPCR Master Mix (Bio-Rad) and forward and reverse primers for the respective genes.

The primers for the target genes were designed using Beacon Designer software (Premier Biosoft, Palo Alto, CA, USA) as shown in Additional file 3: Table S3 and obtained from Integrated DNA Technologies (Coralville, IA, USA). The reaction was performed on a CFX Real time PCR system from Bio-Rad using an optimised protocol [19, 20]. Cycle threshold (Ct) values were obtained, and ΔΔCt values for the experimental and control samples were determined. The relative expression ratios between groups from cycle threshold (Ct) values were determined as described elsewhere [19, 20].

### Data analysis

The quantitative values of factors in medium and transcripts in cells from each culture group with different doses of AMA were log transformed and analysed using the one-way analysis of variance followed by the Tukey’s test and Student’s *t*-test, respectively. Linear regression was done from individual values for obtaining dose dependent profiles for twelve (12) cytokines that appeared important in *post-hoc* analysis. All statistical analyses were done using SPSS v10.0 (SPSS Inc., CA, USA). Significant changes were derived for each bin showing *P* < 0.05.

### Enrichment analysis

For *post hoc* enrichment analysis, candidate cyto/chemokines products were matched with known products into functional ontologies for ‘common’, ‘similar’ and ‘unique’ sets. The probability of a random intersection between a candidate on the target list and ontology entities was estimated in terms of p-values. A lower p-value meant higher relevance of the entity to the data set due to a higher rating for the entity. Enrichment analyses were performed using a cut-off threshold (pFDR(*p*) = 0.05) for the secretory cytokines secreted and showing differential secretion in response to AMA in order to identify the enriched biological pathways and networks. The Analyze Networks (AN) algorithm with default settings was used to retrieve interaction networks that were potentially influenced by AMA. The enrichment analysis and network constructions were achieved using a Metacore bioinformatics platform (GeneGO, St. Joseph, MI, USA) [13, 19, 20].

## Results

**Fig. 1:**
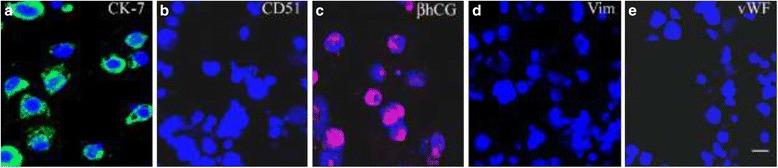
Immunocytochemical characterization of isolated villous cytotrophoblast cells. Isolated cells were allowed to attach to the collagen biomatrix and immunostained for cytokeratin 7 (CK-7; **a** *green*; 97 % positive), vitronectin receptor (CD51; **b** *green*; 95 % negative) and βhCG (**c** *red*; 92 % positive), vimentin (**d** *red*; 99 % negative), vWF (**e** *red*; 99 % negative). Nuclei are counterstained with DAPI (*blue*). Bar = 40 μm

The cell yield was ~3 × 10^6^ cells/g wet weight of placental villous tissue with cell viability in trypan blue exclusion method being more than 92 %. As shown in Fig. 1, these cells were consistently immunopositive for cytokeratin 7 (CK-7, 95 ± 2 %) and βhCG (91 ± 4 %) and negative for vitronectin receptor (CD51; 91 ± 5 %), vimentin (Vim; 98 ± 1 %) and vWF (99 ± 1 %) with overall viability more than 90 % with no significant difference among treatment groups at the time of experiment.

### Profiles of secreted cyto/chemokines

Sixteen (16) cyto/chemokines (CCL7, CCL11, CCL27, IFNG, IL2, IL4, IL5, IL7, IL10, IL15, IL17, LTA, NGFB, PDGFB, SCF and TRAIL) could not be detected in any sample out of the thirteen (13) samples studied. GMCSF, IL3, IL12p40, IL12p70 and IL13 could be detected in six different samples with highly variable dynamic ranges. These twenty one (21) cytokines, therefore, were not subjected to further analysis. Table 1 shows the list of total twenty-seven (27) cytokines which were consistently (>0.1 pg/mg protein in at least 80 % cultures) detected in the conditioned media with isolated cytotrophoblasts. Except three (3) cytokines (IL9, TNF and VEGF), all other cytokines were found to be more than 1 pg/mg protein in the conditioned media.

**Table 1:** Concentration^a^ of consistently^b^ secreted cytokines in conditioned medium of isolated cytotrophoblasts exposed to AMA for 24 h

Cytokine (Alias)	Concentration (ng/ml) of AMA applied				
	0	1	10	100	1000
CCL2 (MCP1)	96.8 (3.2)	80.8 (10.2)	78.7 (14.6)	73.1** (5.2)	74.5** (5.9)
CCL3 (MIP1Α)	49.0 (2.9)	53.2 (5.2)	53.3 (5.2)	44.4 (3.4)	38.5* (2.1)
CCL4 (MIP1B)	298.0 (22.1)	298.3 (12.9)	350.6 (35.2)	265.2* (12.6)	260.2** (12.4)
CCL5 (RANTES)	362.4 (20.7)	367.9 (30.3)	362.4 (21.3)	332.0* (17.9)	309.7* (11.6)
CLEC11A (SCGFB)	592.1 (58.9)	602.9 (72.0)	609.3 (94.9)	559.7 (68.7)	544.9 (68.4)
CXCL1 (GROΑ)	678.0 (61.2)	688.5 (22.9)	649.2 (33.4)	529.4** (29.3)	527.2** (30.6)
CXCL9 (MIG)	113.2 (10.4)	112.9 (16.0)	114.2 (12.6)	97.1 (15.4)	99.4 (17.9)
CXCL10 (IP-10)	152.7 (12.5)	143.7 (14.2)	119.5 (28.0)	129.9 (29.9)	155.21 (10.74)
CXCL12 (SDF-1)	87.5 (14.1)	89.6 (26.7)	99. 9 (27.5)	83.8 (13.3)	79.6 (23.5)
FGF2 (basic FGF)	4.4 (0.3)	4.9 (0.2)	4.8 (0.2)	4.1 (0.9)	3.2* (0.5)
GCSF (CSF-3)	2307.5 (101.6)	2260.1 (138.4)	2242.3 (160.1)	2127.0 (177.3)	1946.0 (250.6)
HGF (SF)	12501.5 (1648.7)	10638.3 (1955.3)	10935.2 (1194.5)	9930.7 (874.5)	9920.1 (834.8)
IFNA2	4.9 (1.9)	11.1 (5.4)	4.5 (0.9)	4.9 (2.9)	4.7 (1.6)
IL1A	113.4 (14.9)	106.0(15.5)	112.8 (17.0)	128.7 (18.2)	120.6 (13.4)
IL1B	353.0 (23.4)	333.1 (34.2)	322.7 (31.6)	313.5* (26.9)	302.2** (10.6)
IL-1RA	32.9 (2.6)	30.2 (6.1)	27.8 (6.5)	29.5 (1.0)	28.8 (3.0)
IL-2RA (CD25)	ND	ND	2.3^*(i)*^ (0.1)	2.2^*(i)*^ (0.1)	2.9^*(i)*^ (0.2)
IL6 (IFNΒ2)	752.7 (41.8)	736.2 (40.1)	704.3 (31.3)	611.5** (22.6)	587.8** (26.1)
IL8 (CXCL8)	11779.0 (2146.6)	12706.4 (2868.5)	16701.7 (4785.2)	14902.2 (4615.2)	8302.9* (2435.9)
IL9 (HP40)	0.6 (0.3)	0.3 (0.2)	0.3 (0.2)	0.4 (0.2)	0.3 (0.2)
IL16 (LCF)	11.4(3.9)	11.0 (4.8)	18.7* (2.4)	18.3* (2.6)	18.8* (2.2)
IL18 (IGIF)	28.9 (2.8)	30.2 (2.3)	28.0 (3.0)	25.2 (1.7)	29.2 (1.7)
LIF (DIA)	2.8 (1.2)	2.9 (1.4)	2.9 (1.3)	2.8 (1.2)	3.1 (1.3)
MCSF (CSF1)	174.2 (16.2)	125.9* (23.6)	123.7* (21.3)	121.6* (27.9)	125.6** (7.4)
MIF (MMIF)	2919.2 (178.5)	2790.7 (211.2)	2708.6 (260.7)	2231.1* (261.3)	2154.2** (143.1)
TNF (TNF alpha)	0.6 (0.3)	0.5 (0.2)	0.4 (0.2)	0.4 (0.2)	0.4 (0.3)
VEGF-A (VPF)	0.3 (0.1)	0.2 (0.1)	0.2 (0.1)	0.2 (0.1)	0.2 (0.1)

Values are shown as means with SDs in *brackets*. ^a^pg/mg of Bradford protein. Note that concentrations were estimated in terms of pg per unit mg of secreted protein (w/w) to avoid any bias in mass per unit volume. ^b^ ≥ 0.1 pg/mg of Bradford protein detected in at least 80 % samples. ^*(i)*^induced. *P < 0.05, **P < 0.01 as compared to control. ND, values <0.1 pg/mg Bradford protein

### Differential cyto/chemokine secretion profile following AMA administration

**Fig. 2:**
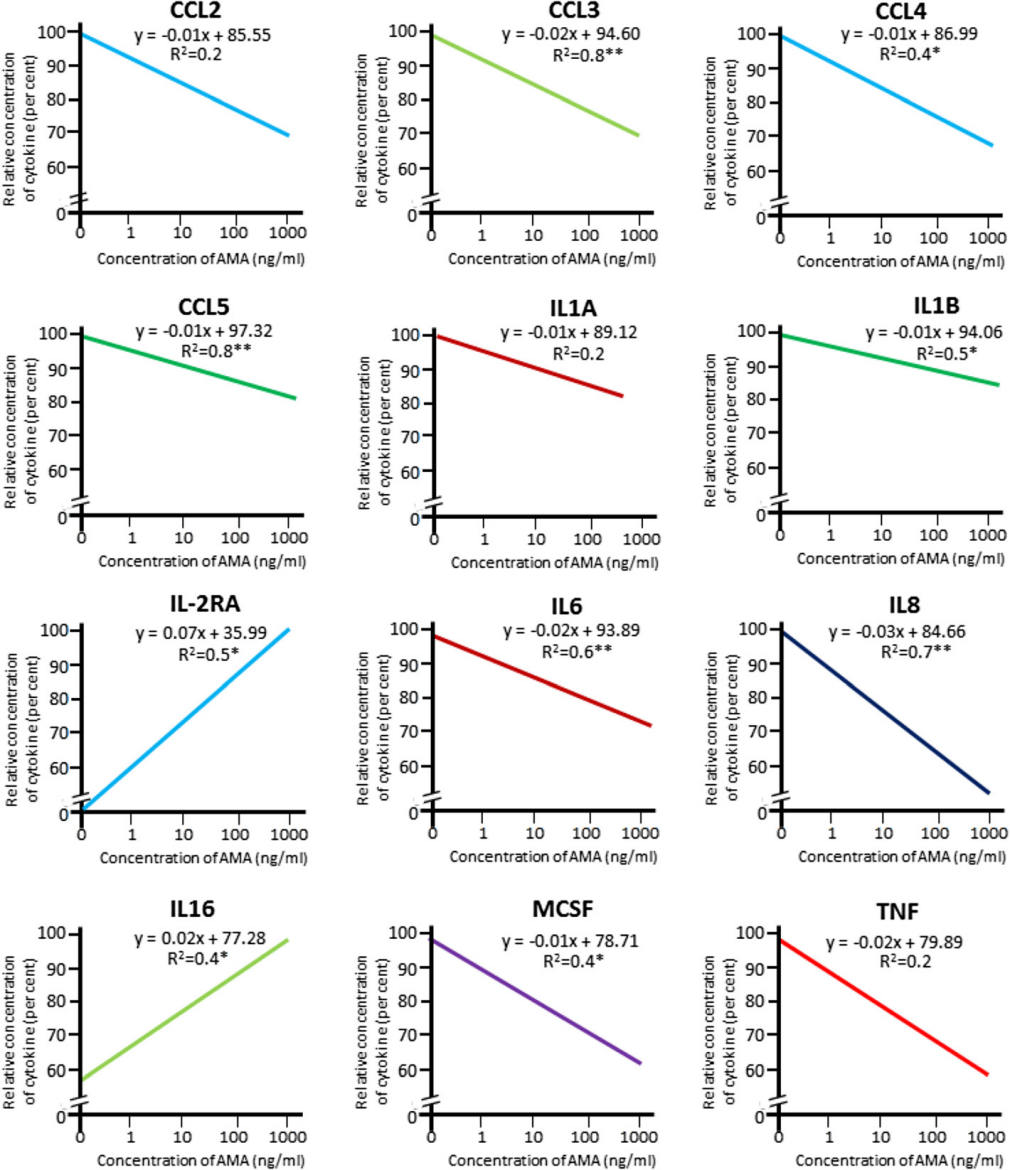
The linearized profiles of putatively important cytokines in the conditioned medium of villous cytotrophoblasts in primary culture following the administration of different doses (0, 1, 10, 100, 1000 ng/ml) of AMA. Linear regressions were obtained from individual values, taking the estimated maximum values as 100 % for obtaining dose-dependent profiles. The twelve (12) cytokines were selected based on enrichment analysis. **P < 0.01. *P < 0.05

Analysis of variance of concentrations of the cytokines consistently detected in conditioned media following different doses of AMA administration identified significant (*P* < 0.05) decrease for seven (7) cytokines (CCL2, CCL4, CCL5, CXCL1, IL1B, IL6, MIF) following the application of 100 and 1000 ng/ml AMA, and additional three (3) cytokines (CCL3, FGF2, IL8) only following the application of 1000 ng/ml AMA as compared to that of the basal control without AMA (Table 1). MCSF (CSF1) showed significant reduction in all doses of AMA as compared to the control samples. IL-2RA was detected in 10–1000 ng/ml AMA treated samples and non-detectable in samples obtained from no AMA and 1 ng/ml AMA groups. Of the affected cytokines, a group of cytokines (CCL2, CCL3, CXCL1, FGF2, IL6, IL8, MCSF, and MIF) showed a decrease of 2030 % and the other affected cytokines (CCL4, CCL5, IL1B) showed a decrease of 10–15 % following the administration of AMA. Only IL16 was higher by about 40 % and IL-2RA was seen to be induced in 10–1000 ng/ml AMA treated groups as compared to other two groups (0 and 1 ng/ml AMA). Based on enrichment analysis, twelve (12) cyto/chemokines from cytotrophoblasts were selected for assessing the linear regression between their concentrations and different doses of AMA. As Fig. 2 shows, it revealed significant dose-dependent correlation for CCL3 (*P* < 0.01), CCL4 (*P* < 0.05), CCL5 (*P* < 0.01), IL1B (*P* < 0.05), IL-2RA (*P* < 0.05), IL6 (*P* < 0.01), IL8 (*P* < 0.01), IL16 (*P* < 0.05) and MCSF (*P* < 0.05). No significant change was seen in the residual fourteen (14) cytokines (CLEC11A, CXCL9, CXCL10, CXCL12, GCSF, HGF, IFNA2, IL1A, IL-1RΑ, IL9, IL18, LIF, TNF, VEGF).

**Fig. 3:**
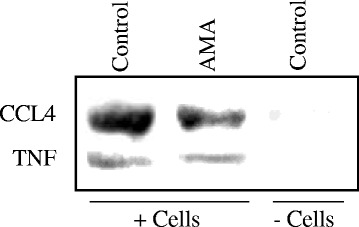
Representative Western immunoblots of CCL4 and TNF from conditioned media without cells (Control, − cells/+AMA), and with cytotrophoblast cells grown in primary culture on collagen biomatrix (+ cells) with AMA (1000 ng/ml) and without AMA (Control, +cells). Media containing 20 μg of Bradford proteins were subjected to electrophoretic separation followed by immunoblot analysis. The log transformed measures of optical densities for each target antigen were calculated as per cent of total integrated optical densities for total protein loaded as described in the Methods section. The values for CCL4 in specific experiment were 11.8 (Control, +cells), 6.7 (AMA, +cells) and 0.7 (Control, −cells/+AMA), and for TNF were 1.6 (Control, +cells), 1.2 (AMA, +cells) and 0.8 (Control, −cells/+AMA) per cent of integrated optical densities, resepectively

As shown in Fig. 3, Western blot analysis revealed about 1.5-fold change (*P* < 0.01) in the level of immunopositive CCL4 between conditioned medium obtained from control cell culture (12.3 ± 2.2) and 1000 ng/ml AMA containing cell culture (7.1 ± 2.1); both were higher (*P* < 0.001) than its level in unconditioned basal medium. On the other hand, immunopositive TNF level was extremely low in the conditioned medium obtained from both control cell culture (2.1 ± 0.6) and 1000 ng/ml AMA culture (1.6 ± 0.5) with statistically no significant difference, although they were marginally higher (*P* < 0.05) than that in basal medium without cells (1.0 ± 0.3) (Fig. 3). Thus, the Western blot analysis revealed vector concordance with their profiles estimated by using multiplex immunoassays technique.

### Steady state transcript levels of differentially secreted cytokines

Table 2 shows the comparative profiles of Bio-Plex cytokine data and real time RT-PCR data for nine candidates (CCL4, CCL5, IL1A, IL1B, IL-2RA, IL6, IL16, MIF and TNF) based on protein data as seen in Table 1 and the results of the enrichment analysis (as given in Table 3) in five samples treated without or with (1000 ng/ml) AMA from original set of thirteen samples. In addition to IL1A and TNF which showed no change in the secretory concentrations as welll, no change was observed in the transcript profiles in any of those cytokines [CCL4 (MIP1B), CCL5 (RANTES), IL1B and IL6] except MIF which showed significant decrease in their secreted concentrations following AMA treatment (Table 2). However, the steady state transcript levels of IL-2A and IL16, which showed significantly higher secretion following AMA treatment, were also significantly higher in cells treated with AMA as compared to cells in control culture (Table 2).

**Table 2:** Comparative profiles of immunopositive proteins in medium and respective transcripts in cells for selected cytokines in primary cultures with (1000 ng/ml) and without AMA

Name of cytokine	Mean ± SD (*n* = 5/ea)			
	Concentration in conditioned medium^a^		Relative level of transcript in cells^b^	
	Control	AMA	Control	AMA
CCL4 (MIP1B)	298.0	260.2**	2.5	2.6
	±22.1	±12.4	±0.7	±0.9
CCL5 (RANTES)	362.4	309.7*	5.2	5.8
	±20.7	±11.6	±2.7	±2.1
IL1A	113.4	120.6	2.9	3.6
	±14.9	±13.4	±2.4	±2.1
IL1B	353.0	302.2**	2.5	2.9
	±23.4	±10.6	±1.1	±1.5
IL-2RA	0.08^c^	2.9***	7.3	9.5**
	±0.03	±0.1	±0.1	±0.3
IL6	752.7	587.8**	5.8	6.9
	±41.8	±21.1	±1.2	±1.8
IL16	11.4	18.8*	7.5	8.8**
	±3.9	±2.2	±0.1	±0.2
MIF	2919.2	2154.2	9.8	8.3*
	±178.5	±143.0	±0.6	±0.4
TNF	0.6	0.4	3.0	3.1
	±0.3	±0.3	±0.5	±0.8

^a^pg per mg Bradford protein from Bio-plex immunoassays and as shown in Table 1. ^b^ΔΔC_t_ relative to housekeeping genes (GAPDH and UBC) based on real time RT-PCR using SYBR green uniplex chemistry. *P < 0.05, **P < 0.01, ***P < 0.001 as compared to control. ^c^values <0.1 pg/mg Bradford protein considered not detectable

**Table 3:** Summary of top-scored features from reports of enrichment and pathways-networks analysis

Analysis	Description of the process (*p* value)	Candidates^d^
Gene ontology (GO) cellular process^a^	Inflammatory response (5.9E-12)	*CCL3*, *CCL4*, *CCL5*, *CXCL1*, IL1A, *IL1B*, *IL6*, IL9, IL18, IL-1RA, **IL-2RA,** CXCL9, CXCL10, *MIF*, TNF
	Immune response (6.3E-12)	*CCL3*, *CCL4*, *CCL5*, G-CSF, *CXCL1*, CXCL10, IL1A, *IL1B*, *IL6*, IL9, IL18, IL1RA, **IL-2RA**, LIF, *MIF*, TNF, VEGF
	Regulation of cell proliferation (6.3E-12)	*CCL3*, *CCL4*, *CCL5*, HGF, GCSF, *CXCL1*, CXCL9, CXCL10, IL1A, *IL1B*, *IL6*, IL9, IL18, IL-1RA, **IL-2RA**, LIF, *MIF*, TNF, VEGF
	Response to wound healing (6.4E-12)	*CCL3*, *CCL4*, *CCL5*, *CXCL1*, CXCL9, CXCL10, HGF, IL1A, *IL1B*, *IL6*, IL9, IL18, IL-1RA, *MIF*, TNF, VEGF
	Cell chemotaxis (6.5E-12)	CLEC11A, *CCL4*, *CCL5*, *CXCL1,* CXCL9, CXCL10, HGF, *IL1B*, *IL6*, **IL-2RA**, VEGF
Enrichment by pathways map^b^	Cyto/chemokines in inflammation (3.8E-12)	*CCL4, CCL5,* CXCL10*,* IL1A, *IL1B, IL6,* IL9*,* TNF
	Secreted signals in immune response (3.9E-12)	*CCL4, CCL5,* CXCL10*,* IL1A, *IL1B*, IL-1RA, **IL- 2RA**, *IL6,* IL9, TNF
Most relevant network^c^	Toll-like receptor signaling pathway (2.3E-12)	IL1A, *IL1B, ΙL6, MIF,* TNF

^a^sources: ebi.ac.uk/quickGO/, Portal.genego.com/cgi/, and Amigo.geneontology.org/amigo

^b^top scored pathways were identified using input list of selected candidates showing differential levels on Metacore GeneGO portal (MetaCore Biomanual 5.0. at https://portal.genego.com/help/MetaCpre_bio_manual_5) as discussed elsewhere [11, 17, 18]. Source: portal.genego.com/

^c^The cyto/chemokines displaying differential secretion following administration of AMA as shown in Table 1 used as the input list for generation of biological networks using Analyze Networks (AN) algorithm with default settings. This is a variant of the shortest paths algorithm with main parameters of (i) relative enrichment with the uploaded data from Table 1 and (ii) relative saturation of networks with canonical pathways. These networks are built on the fly and unique for the uploaded data. In this workflow the networks are prioritized based on the number of fragments of canonical pathways on the network as discussed elsewhere [17, 18]. Source: Portal.genego.com/

^d^Cyto/chemokines detected in all groups. The negatively affected candidates by AMA are shown in *italics* and positively affected ones are shown in **bold**

### Enrichment analysis

**Fig. 4:**
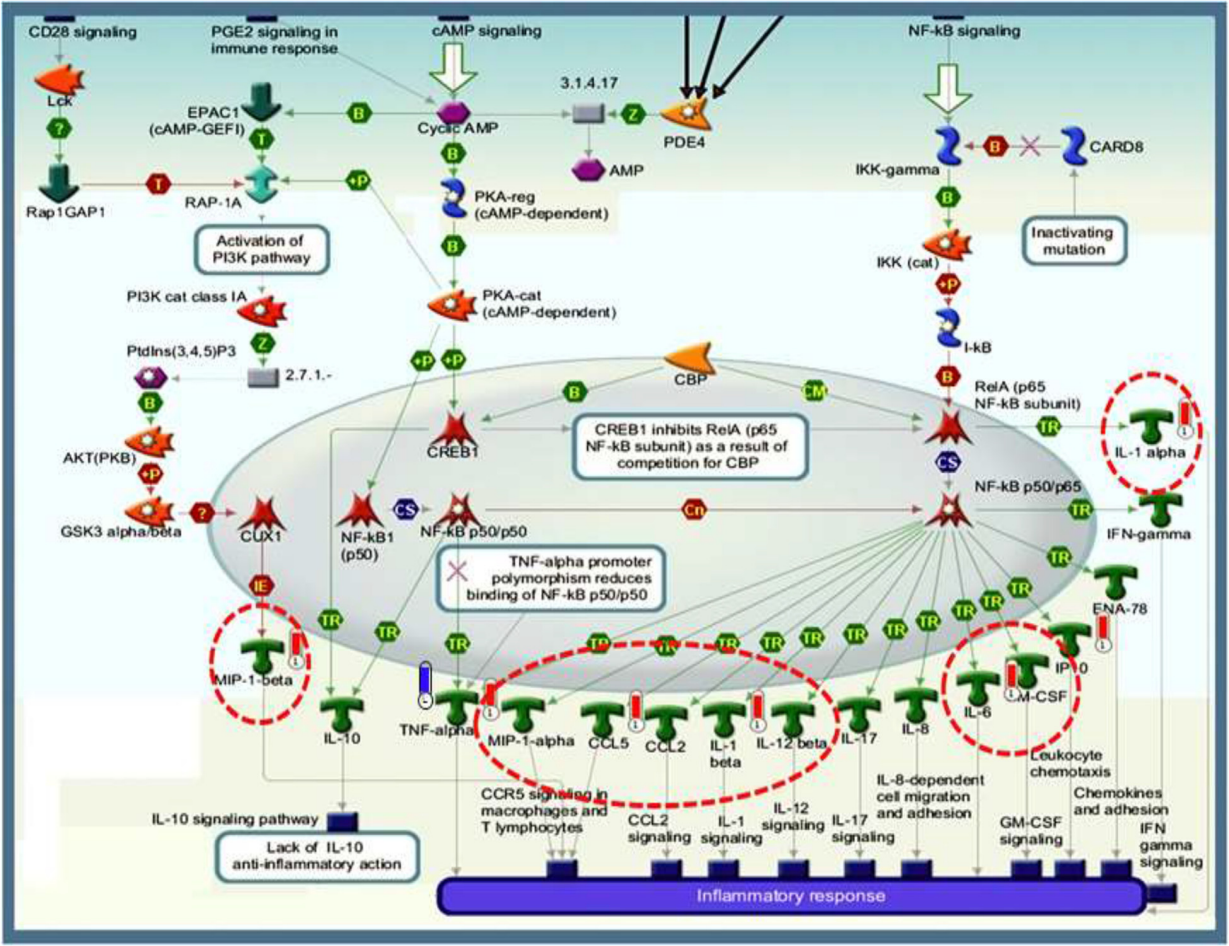
A snapshot of the top-scored pathway map based on the enrichment distribution sorted by ‘Statistically Significant Maps’ set using Metacore portal. This pathway map was retrieved from the significant pathways involved in inflammatory responses. Experimental data from all files is linked to and visualized on the maps as thermometer like figures (*red*, down-regulated signals; *blue*, no change) to indicate vector component of the cytokine expression in the treatment groups. The cluster of cyto/chemokines which were markedly affected by AMA in the present study are shown in enclosures with broken red lines. B, binding; CM, covalent modification; Cn, competition; CS, complex subunit; IE, influence on expression; +P, phosphorylation; T, transformation; TR, transcription regulation; Z, catalysis. *Green* denotes positive effect. *Red* denotes negative effect

Enrichment analysis based on input list of cyto/chemokines secreted by cytotrophoblasts identified that the integral modules of inflammatory and immune responses were potentially involved in the target cells and were affected by the administration of AMA. Figure 4 shows a snapshot of inflammatory-immunomodulatory pathway map involving the cyto/chemokines secreted from early placental villous cyto/chemokines and the ones which were affected by AMA. Enrichment analysis also revealed that other important cellular modules related to regulation of cellular proliferation, response to wound healing and cell chemotaxis were putatively influenced by the cyto/chemokines secreted by cytotrophoblasts, and were affected by the administration of AMA. Table 3 shows the summary of the significant outputs of various enrichment analyses for cyto/chemokines showing differential secretion between samples with and without AMA. It is notable that mediators of Toll-like receptors (TLRs) signaling pathway involving IL1, IL6, MIF, and TNF was identified in the enrichment analysis of cyto/chemokines secreted by cytotrophoblasts and those which were affected by the administration of AMA (Table 3).

## Discussion

**Fig. 5:**
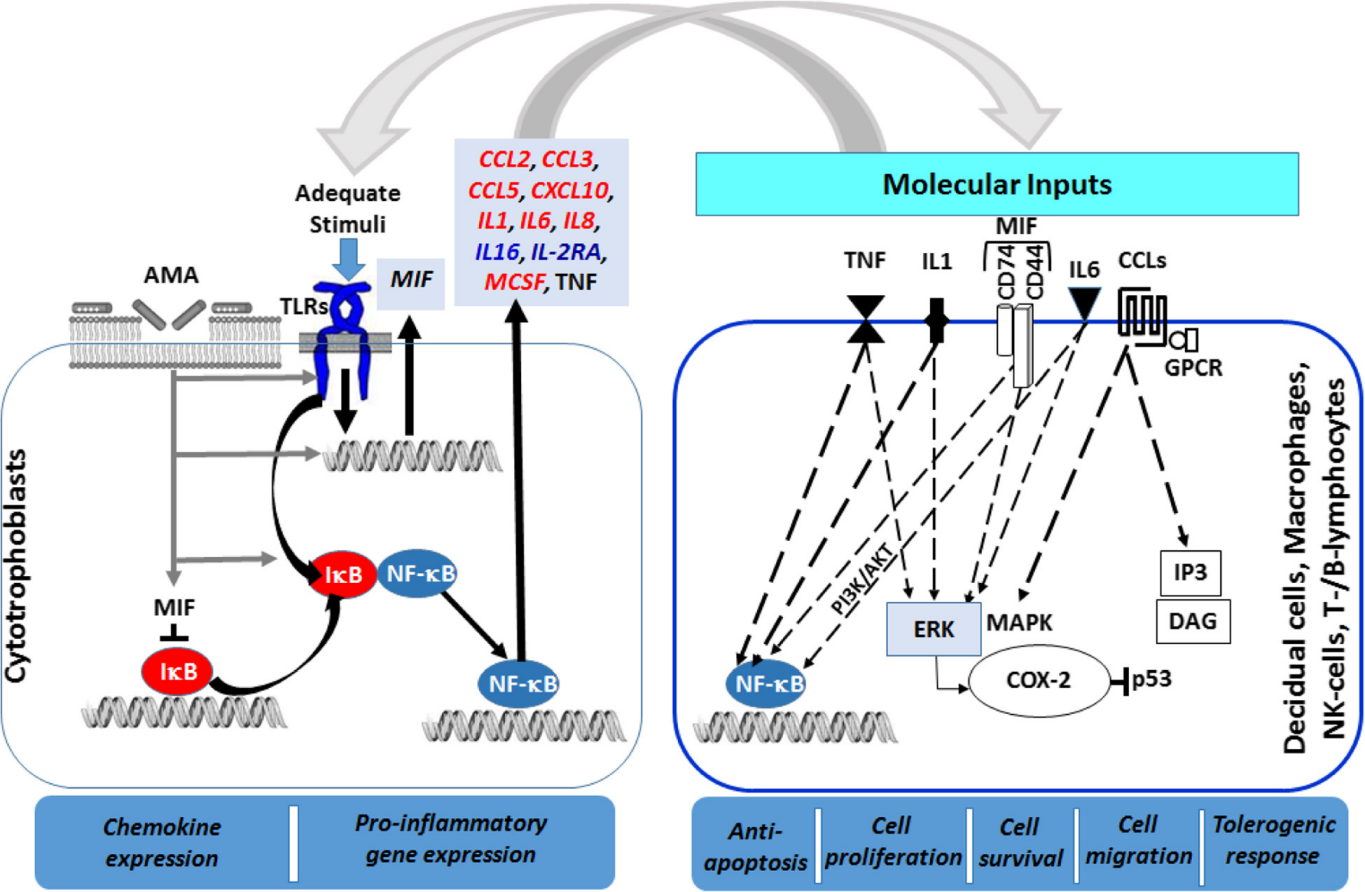
Schematic presentation of a model highlighting the probable mode of action of AMA on placental development. Typically, Toll-like receptors (TLRs) mediated action in cytotrophoblasts under the influence of adequate stimuli from decidual cells, T-cells, B-cells, macrophages and NK cells at maternal-fetal interface results in elaboration of a group of cyto/chemokines (CCL2, CCL3, CCL5, CXCL10, IL1, IL6, IL16, IL-2RA, MIF and TNF) which are associated with inflammatory and tolerogenic activities. Substantial evidence suggests that Toll-like receptor signaling pathway involving IL1, IL6, and TNF is critical during early placental development. As shown, many of these cytokines via specific receptors and associated molecules with down-stream engagement of effector moieties (e.g., NF-kB, ERK, MAPK, IP3, DAG and p53) regulate different cellular processes (e.g., inflammatory responses, cell survival, growth, proliferation, migration and apoptosis) on homotypic and heterotypic cells as shown by *dashed arrows*. The helical peptide like AMA with cationic moieties negatively affects the above-mentioned process at multiple levels as shown by *gray arrows*. Thus, AMA peptides putatively affect a variety of different cellular outcomes, depending upon the peptide concentrations and the nature of its interactions with cell membranes and membrane proteins, resulting in inadequacy of cellular homeostasis. The cytokines upregulated by AMA are shown in *blue italics* and down-regulated ones by AMA are shown in *red italics*. For further details, see the Discussion section and relevant references [12, 19, 21–32, 45–47, 49, 65, 69–71]

The natural interaction between embryonic trophoblasts and maternal uterine cells during blastocyst implantation and placentation involves several cytokines; the underlying process in many ways is physiognomonic of an inflammatory state [21, 22]. It is generally considered that cytokines are produced by immune competent cells. However, it now appears that various pro- and anti-inflammatory cytokines are also synthesized and secreted by placental trophoblasts [23, 24]. The results of the present study, in which we have used a panel of 48 cytokines, chemokines and growth factors, indeed substantiates and widens our information in this regard. The observations from the present study that human villous cytotrophoblasts isolated from first trimester placental villi and grown on collagen biomatrix elaborated 27 cytokines reportedly involved in various cellular physiology and pathology [25–28], and that secretion of thirteen (13) out of 27 cytokines were affected by a model synthetic cationic antimicrobial peptide (CAMP), i.e., AMA along with previous reports [21, 23, 24, 29, 30] open up a new vista for further investigation into the possible impact of CAMPs on first trimester placental biology [31, 32]. It appears that the cyto/chemokines and growth factors that were identified in the conditioned medium of cytotrophoblasts and the ones which were affected by AMA are generally known to influence several cellular processes and pathways ranging from inflammatory responses to immune competence, regulation of cellular proliferation to cell chemotaxis (Table 3, Fig. 5).

### Cytokines elaborated by early placental cytotrophoblasts

It is evident from the present investigation that a large number of cytokines were released by villous cytotrophoblasts. Earlier Lash and associates have reported the presence of some of these cytokines (IL1B, IL8 and CCL1/MCP1) in multiplex analysis for a dozen of cytokines (IL1B, IL2, IL4, IL5, IL8, IL10, IL12p70, IL13, IFNG, GMCSF, MCP1 and RANTES) of the conditioned media of first trimester placental villous and extravillous cytotrophoblasts [24]. Interestingly, IL12p70 and IL13 were detected in media conditioned with first trimester villous cytotrophoblasts in the previous study [24], whereas we found that these two interleukins were detectable only in 45 % samples and were secreted at highly variable dynamic ranges; hence these two interleukins were considered inconsistent in the present study. On the other hand, CCL5 (RANTES) which was consistently detected in conditioned media of villous cytotrophoblasts in the present study, was not detected in the previous study [24]. The observed differences might have resulted from differences in the details of cell culture protocols used in these two studies. For example, in the present study the culture medium used was serum-free, but supplemented with insulin, transferrin, selenium and hydrocortisone and cells were grown on collagen I biomatrix, whereas in the previous study 10 % fetal bovine serum was used and cells were grown on fibronectin coated substratum [24]. Whether these variables could indeed have resulted in the observed differences is only conjectural at this point. However, it is known that differences in the traction forces generated by the two different extracellular matrix molecules, namely collagen and fibronectin may differentially influence gene and protein expressions in cells [33].

### AMA affected cytokine secretion by early placental cytotrophoblasts

The various cyto/chemokines which were seen to be decreased in the conditioned media of isolated cytotrophoblasts by the application of AMA included CCLs-2 (MCP1), 3 (MIP1A), 4 (MIP1B), 5 (RANTES), CXCLs-1 (GROA) and 8 (IL8); these are known to function in combinatorial manner towards regulating inflammation, chemotactic movements of pro-inflammatory cells, cell proliferation and wound healing [34–37].

The growth factors like FGF2 and CSF1 which are known to support the growth and differentiation of placental trophoblasts [38–40] were also down-regulated by AMA. Collectively, our results provide probable physiological basis of our earlier observation that application of AMA resulted in the breakdown of cellular homeostasis in isolated first trimester villous cytotrophoblasts [9, 10].

We have also observed a relative lowering of secretion IL1B, IL6 and MIF with no change in the level of secretory TNF along with increased transcript levels and secretory concentrations of IL16 and IL-2RΑ by isolated cytotrophoblasts following AMA treatment in vitro. There is substantial evidence that the shift in the ratios of these inflammatory mediators may result in differential status of oxidative stress, cell proliferation and apoptosis, NF-kB recruitment and down-stream signaling, and regulation of activation of cytoplasmic ‘death domain’ in cell specific manner [26, 41–43].

Interestingly, it was observed that AMA did not affect the steady state transcript levels of the candidate cyto/chemokines (CCL4, CCL5, IL1A, IL1B and IL6) in the cytotrophoblasts, the secreted levels of which were reduced by in vitro administration of AMA. It may be speculated that AMA did not change the steady state transcript profiles of those cyto/chemokines which however displayed reduced secretion by its action, possibly on translational, post-translational and secretory processes. Furthermore, the fact that AMA itself is an anti-microbial agent and that it down-regulates immune responsiveness in placenta might be viewed as the resultant steady state of intrinsic controlled output of the system [44]. This suggestion appears to be probable on teleological ground, however, further studies are necessary to understand the underlying molecular mechanism of action of CAMPs in this regard.

### AMA affected secretion of cytokines by cytotrophoblasts in differential manner

The results of the present study revealed an interesting differential effect of AMA on cytokine secretion and the steady state profiles their respective cytokines by cytotrophoblasts in vitro, indicating that the observed effects of AMA on cytotrophoblasts was not a general one, rather specific for dose of AMA for individual cytokines. While the secretion of a few cytokines (CCL2, CCL4, CCL5, CXCL1, IL1B, IL6, and MIF) was significantly reduced by AMA at 100 and 1000 ng/ml, and some (CCL3, FGF2, and IL8) only at 1000 ng/ml, MCSF (CSF1) showed significant reduction in all doses of AMA applied. IL16 and IL-2RA were significantly higher at 10–1000 ng/ml. Although it signifies that AMA at 1 ng/ml could affect the cellular physiology of cytotrophoblasts, only little dose-dependent changes were observed with clear effect for various cytokines at 100–1000 ng/ml AMA, and there were also innate differences in responses of secretion of cytokines with some degree of specificity in the cytotrophoblasts in primary culture. The underlying physiological significance and mechanism of these observations of dose-differential effect of AMA on cytotrophoblasts are only conjectural at present. However, it brings the issue of physiological concentration of CAMP in the female reproductive tract against the administered amount of AMA in the present study.

The minimum inhibitory concentrations of CAMP with broad spectrum of antimicrobial activity against gram-positive and gram-negative bacteria, fungi and protozoa can be as low as 0.4 μM [45]. The vaginal concentration of gene-encoded CAMP, on the other hand, may rise to as high as 10 μM [46]. It is notable that the maximum concentration of AMA used in the present study was 0.4 μM. Thus, apparently the dose of AMA used was well within the normal level. Nevertheless, we believe that such generalization needs to be taken with due caution. For example, AMA is inherently highly stable synthetic compound because of alanine replacement in 8,13 and 18 positions and its amide residue in the terminus [8]. Also, AMA administered in the culture medium by design yields a closed and limited compartmental distribution pharmacokinetic system in contrast to CAMP inherently present in the tissue itself in a living organism yielding an open and multi-compartmental pool system. Further experiments are indeed necessary to retrieve the therapeutic correspondence of synthetic CAMP in the intact system using appropriate model systems [47].

### Significance of CAMP action in placental development

The maternal-fetal interface is one of the ‘immune privileged sites’ where active mechanisms of acquired immunity are muted or bypassed as an adaptive function in the process of evolution of mammalian placenta. We have earlier reported that first trimester villous cytotrophoblasts drive relative up-regulation of innate antiviral response and down-regulation of adaptive immune response as the first site of defense [20, 48]. This along with the immune functioning of decidual cells, T-cells, B-cells, macrophages and NK cells hold the immune privileged status at maternal-fetal interface [49]. Substantial evidence suggests that Toll-like receptor signaling pathway involving IL1, IL6, and TNF is critical for initiating inflammatory activation in response to cellular stress [26–28] and during early placental development [21, 23, 24, 29–32]. Toll-like receptor signaling pathways also involve macrophage migration inhibitory factor (MIF) which exerts pleotropic action on several cellular processes including cell survival, apoptosis and inflammation [50–52]. Primary trophoblast cells of human placenta suggestively function in a regulated fashion through the activation of Toll-like receptors (TLRs) [30, 53].

Figure 5 shows a scheme modeling how Toll-like receptors signaling may influence the cellular physiology of placental trophoblasts, and how AMA may affect Toll-like receptor signaling pathway in first trimester villous cytotrophoblasts causing changes in ratios of different secretory pro-inflammatory cyto/chemokines which affects the tissue homeostasis by disturbing its functional synchrony with decidual cells, macrophages and other immune competent cells involved in placental development [54]. Although we have not categorically examined the mechanism of action of AMA on the cytoptrophoblasts in the present study, there is substantial evidence to suggest that some CAMPs can affect signaling pathways of TLRs at several levels from receptors, downstream signal transduction, activation of NF-kB factors and genetic levels, collectively resulting in inhibition of the expression of specific proinflammatory factors which are generally upregulated by NF-kB [55–57].

The physiological significance of the increased expression and secretion of IL16 and IL-2RΑ in cytotrophoblasts following application of AMA is not clear. IL16 is a pleotropic cytokine which can influence chemotaxis, immune and inflammatory responsiveness, cellular physiology and transcriptional regulation in various cells [58–62]; it is known to induce IL-2RΑ (CD25) expression [63] and functions synergistically with IL2 to prime T-cell proliferation [64]. It appears possible that secretory IL16 and IL-2RΑ (CD25) play significant role in initiating a tolerogenic profile in the decidua through the recruitment of specific subpopulation of CD4^+^ CD25^+^Foxp3^+^ regulatory T-cells [65]. It has been suggested that increased secretory mature form of IL16 production by trophoblasts contributes to the altered balance in the immune-inflammatory responses often associated with pregnancy disorder [66]. The immunological and inflammatory regulation mechanisms of the placental environment are altered in recurrent miscarriage compared with control along with significant up-regulation of trophoblast mRNA for CD-25 (IL-2RA) [67]. Thus, it appears plausible based on above-mentioned studies that the increased secretion of these two interleukins (IL16, IL-2RA) and decreased secretion of IL1, IL6 and MIF by cytotrophoblasts following the application of AMA exerts combinatorial effect intervening the balance of the overall immune-inflammatory status of the trophoblast bed at steady state. It is notable in this connection that these cytokines and TLRs are intimately involved in the process of innate immunity and resistance to tolerogenesis in allotransplantation [68]. Coolectively, it appears that CAMPs mediated derangement in the profile of cyto/chemokines in early placental cytotrophoblasts may interfere with the normal immunomodulatory and other functions of placenta [69–71].

Finally, experiments in the present study were performed in vitro using isolated villous cytotrophoblasts from placental samples, and therefore, the interpretation of the results is limited and tentative. Further verification of functions of placental trophoblasts and pregnancy outcome upon administration CAMP is necessary using non-human primate models.

## Conclusion

In conclusion, it appears that early placental villous cytotrophoblasts secrete a host of cytokines, many of which appear to be affected by the administration of a synthetic CAMP, Ala^8,13,18^-magainin II amide (AMA). Based on previous observations that AMA has anti-nidatory action and that it has untoward action on cellular homeostasis in early placental cytotrophoblast, it may be conjectured from the results of the present study that AMA mediated derangement in the profile of cyto/chemokines in early placental cytotrophoblasts may interfere with the normal immunomodulatory and other functions of placenta.

## Additional files

Additional file 1: Table S1. Cytokines, chemokines and growth factors studied. (DOCX 18 kb) Additional file 2: Table S2. Characteristics of targets and primary antibodies used in the study. (DOCX 19 kb) Additional file 3: Table S3. List of genes and their primers used for quantitative real time RT-PCR. (DOC 34 kb)

## References

[1] Hancock RE, Sahl HG (2006). Antimicrobial and host-defense peptides as new anti infective therapeutic strategies. Nat Biotechnol.

[2] Matsuzaki K, Murase O, Fujii N, Miyajima K (1996). An antimicrobial peptide, Magainin 2, induced rapid flip-flop of phospholipids coupled with pore formation and peptide translocation. Biochemistry.

[3] Sato H, Feix JB (2006). Peptide – membrane interactions and mechanisms of membrane destruction by amphipathic α-helical antimicrobial peptides. Biochim Biophys Acta.

[4] Yeaman MR, Yount NY (2003). Mechanisms of antimicrobial peptide action and resistance. Pharmacol Rev.

[5] Epand RM, Epand RF (2011). Bacterial membrane lipids in the action of antimicrobial agents. J Pept Sci.

[6] Huppertz B, Frank HG, Reister F, Kingdom J, Korr H, Kaufmann P (1999). Apoptosis cascade progresses during turnover of human trophoblast: analysis of villous cytotrophoblast and syncytial fragments in vitro. Lab Invest.

[7] Zasloff M, Martint B, Chen HC (1988). Antimicrobial activity of synthetic magainin peptides and several analogues. Proc Natl Acad Sci U S A.

[8] Chen HC, Brown JH, Morell JL, Huang CM (1988). Synthetic magainin analogues with improved antimicrobial activity. FEBS Lett.

[9] Dhawan L, Ghosh D, Lalitkumar PG, Sharma DN, Lasley BL, Overstreet JW (2000). Anti-nidatory effect of vaginally administered (Ala^8,13,18^)-magainin II amide in the rhesus monkey. Contraception.

[10] Ghosh D, Dhawan L, Lalitkumar PG, Wong V, Rosario JF, Hendrickx AG (2001). Effect of vaginally administered (Ala^8,13,18^)-magainin II amide on the morphology of implantation stage endometrium in the rhesus monkey (*Macaca mulatta*). Contraception.

[11] Lamba P, Kar M, Sengupta J, Ghosh D (2005). Effect of (Ala^8,13,18^) – magainin II amide on human trophoblast cells in vitro. Indian J Physiol Pharmacol.

[12] Sengupta J, Khan MA, Huppertz B, Ghosh D (2011). In-vitro effects of the antimicrobial peptide Ala8, 13, 18 – magainin II amide on isolated human first trimester villous trophoblast cells. Reprod Biol Endocrinol.

[13] Srivastava A, Sengupta J, Kriplani A, Roy KK, Ghosh D (2013). Profiles of cytokines secreted by isolated human endometrial cells under the influence of chorionic gonadotropin during the window of embryo implantation. Reprod Biol Endocrinol.

[14] Malhotra N, Srivastava A, Rana H, Bahadur A, Sengupta J, Ghosh D (2013). Comparative multiplex analysis of cytokines, chemokines and growth factors in follicular fluid of normoresponder women undergoing ovum donation with gonadotropin-releasing hormone agonist versus gonadotropin-releasing hormone antagonist protocols. J Hum Reprod Sci.

[15] Tarrade A, Lai Kuen R, Malassiné A, Tricottet V, Blain P, Vidaud M (2001). Characterization of human villous and extravillous trophoblasts isolated from first trimester placenta. Lab Invest.

[16] de Jager W, Rijkers GT (2006). Solid-phase and bead-based cytokine immunoassay: a comparison. Method.

[17] Walker JM (2002). The protein protocols handbook.

[18] Bustin SA, Benes V, Garson JA, Hellemans J, Huggett J, Kubista M (2009). The MIQE Guidelines: Minimum Information for Publication of Quantitative Real-Time PCR Experiments. Clin Chem.

[19] Khan MA, Sengupta J, Mittal S, Ghosh D (2012). Genome-wide expressions in autologous eutopic and ectopic endometrium of fertile women with endometriosis. Reprod Biol Endocrinol.

[20] Khan MA, Manna S, Malhotra N, Sengupta J, Ghosh D (2014). Expressional regulation of genes linked to immunity & programmed development in human early placental villi. Indian J Med Res.

[21] van Mourik MSM, Macklon NS, Heijnen CJ (2009). Embryonic implantation: cytokines, adhesion molecules, and immune cells in establishing an implantation environment. J Leukoc Biol.

[22] Sengupta J, Ghosh D (2014). Multi-level and multi-scale integrative approach to the understanding of human blastocyst implantation. Prog Biophys Mol Biol.

[23] Bowen JM, Chamley L, Mitchell MD, Keelan JA (2002). Cytokines of the placenta and extra-placental membranes: biosynthesis, secretion and roles in establishment of pregnancy in women. Placenta.

[24] Naruse K, Innes BA, Bulmer JN, Robson SC, Searle RF, Lash GE (2010). Secretion of cytokines by villous cytotrophoblast and extravillous trophoblast in the first trimester of human pregnancy. J Reprod Immunol.

[25] Feghali CA, Wright TM (1997). Cytokines in acute and chronic inflammation. Front Biosci.

[26] Dinarello CA (2000). Proinflammatory cytokines. Chest.

[27] Feldmann M, Saklatvala J, Oppenheim JJ, Feldmann M (2001). Proinflammatory cytokines. Cytokine reference.

[28] Takeuchi O, Akira S (2010). Pattern recognition receptors and inflammation. Cell.

[29] McEwan M, Lins RJ, Munro SK, Vincent ZL, Ponnampalam AP, Mitchell MD (2009). Cytokine regulation during the formation of the fetal-maternal interface: focus on cell-cell adhesion and remodelling of the extra-cellular matrix. Cytokine Growth Factor Rev.

[30] Tangerås LH, Stødle GS, Olsen GD, Leknes AH, Gundersen AS, Skei B (2014). Functional Toll-like receptors in primary first-trimester trophoblasts. J Reprod Immunol.

[31] Wang B, Koga K, Osuga Y, Cardenas I, Izumi G, Takamura M (2011). Toll-like receptor-3 ligation-induced indoleamine 2, 3-dioxygenase expression in human trophoblasts. Endocrinology.

[32] Anton L, Brown AG, Parry S, Elovitz MA (2012). Lipopolysaccharide induces cytokine production and decreases extravillous trophoblast invasion through a mitogen-activated protein kinase-mediated pathway: possible mechanisms of first trimester placental dysfunction. Hum Reprod.

[33] Legant WR, Chen CS, Vogel V (2012). Force-induced fibronectin assembly and matrix remodeling in a 3D microtissue model of tissue morphogenesis. Integr Biol (Camb).

[34] Engelhardt E, Toksoy A, Goebeler M, Debus S, Bröcker EB, Gillitzer R (1998). Chemokines IL-8, GROalpha, MCP-1, IP-10, and Mig are sequentially and differentially expressed during phase-specific infiltration of leukocyte subsets in human wound healing. Am J Pathol.

[35] Fraccaroli L, Alfieri J, Larocca L, Calafat M, Mor G, Leirós CP (2009). A potential tolerogenic immune mechanism in a trophoblast cell line through the activation of chemokine-induced T cell death and regulatory T cell modulation. Hum Reprod.

[36] Grasso E, Paparini D, Hauk V, Salamone G, Leiros CP, Ramhorst R (2014). Differential migration and activation profile of monocytes after trophoblast interaction. PLoS ONE.

[37] Tang SK, Knobloch RA, Maucksch C, Connor B (2014). Redirection of doublecortin-positive cell migration by over-expression of the chemokines MCP-1, MIP-1α and GRO-α in the adult rat brain. Neuroscience.

[38] Saito S, Saito M, Enomoto M, Ito A, Motoyoshi K, Nakagawa T (1993). Human macrophage colony-stimulating factor induces the differentiation of trophoblast. Growth Factor.

[39] Omigbodun A, Coukos G, Ziolkiewicz P, Wang C, Coutifaris C (1998). Macrophage-colony stimulating factor (M-CSF) regulates the expression of fibronectin and its α5 integrin receptor in human trophoblasts. Endocrinology.

[40] Ezashi T, Telugu BP, Roberts RM (2012). Model systems for studying trophoblast differentiation from human pluripotent stem cells. Cell Tissue Res.

[41] Kai H, Kitadai Y, Kodama M, Cho S, Kuroda T, Ito M (2005). Involvement of proinflammatory cytokines IL-1beta and IL-6 in progression of human gastric carcinoma. Anticancer Res.

[42] Scheller J, Chalaris A, Schmidt-Arras D, Rose-John S (1813). The pro- and anti-inflammatory properties of the cytokine interleukin-6. Biochim Biophys Acta.

[43] Hahn WS, Kuzmicic J, Burrill JS, Donoghue MA, Foncea R, Jensen MD (2014). Proinflammatory cytokines differentially regulate adipocyte mitochondrial metabolism, oxidative stress, and dynamics. Am J Physiol Endocrinol Metab.

[44] Schaber J, Lapytsko A, Flockerzi D (2014). Nested autoinhibitory feedbacks alter the resistance of homeostatic adaptive biochemical networks. J R Soc Interface.

[45] Hancock REW, Lehrer R (1998). Cationic peptides: a new source of antibiotics. Trends Biotechnol.

[46] Sorensen OE, Gram L, Johnsen AH, Andersson E, Bangsboll S, Tjabringa GS (2003). Processing of seminal plasma hCAP-18 to ALL-38 by gastricsin: a novel mechanism of generating antimicrobial peptides in vagina. J Biol Chem.

[47] Nielsen EI, Friberg LE (2013). Pharmacokinetic-Pharmacodynamic Modeling of Antibacterial Drugs. Pharmacol Rev.

[48] Huppertz B, Ghosh D, Sengupta J (2014). An integrative view on the physiology of human early placental villi. Prog Biophys Mol Biol.

[49] Bulmer JN, Williams PJ, Lash GE (2010). Immune cells in the placental bed. Int J Dev Biol.

[50] Leng L, Bucala R (2006). Insight into the biology of macrophage migration inhibitory factor (MIF) revealed by the cloning of its cell surface receptor. Cell Res.

[51] Flaster H, Bernhagen J, Calandra T, Bucala R (2007). The macrophage migration inhibitory factor-glucocorticoid dyad: regulation of inflammation and immunity. Mol Endocrinol.

[52] Li X, Jiang S, Tapping RI (2010). Toll-like receptor signaling in cell proliferation and survival. Cytokine.

[53] Abrahams VM, Schaefer TM, Fahey JV, Visintin I, Wright JA, Aldo PB (2006). Expression and secretion of antiviral factors by trophoblast cells following stimulation by the TLR-3 agonist, Poly(I : C). Hum Reprod.

[54] Du MR, Wang SC, Li DJ (2014). The integrative roles of chemokines at the maternal-fetal interface in early pregnancy. Cell Mol Immunol.

[55] Mookherjee N, Brown KL, Bowdish DM, Doria S, Falsafi R, Hokamp K (2006). Modulation of the TLR-mediated inflammatory response by the endogenous human host defense peptide LL-37. J Immunol.

[56] Brown KL, Poon GF, Birkenhead D, Pena OM, Falsafi R, Dahlgren C (2011). Host defense peptide LL-37 selectively reduces proinflammatory macrophage responses. J Immunol.

[57] Alalwani SM, Sierigk J, Herr C, Pinkenburg O, Gallo R, Vogelmeier C (2010). The antimicrobial peptide LL-37 modulates the inflammatory and host defense response of human neutrophils. Eur J Immunol.

[58] Cruikshank WW, Kornfeld H, Center DM (2000). Interleukin-16. J Leukoc Biol.

[59] Wilson KC, Center DM, Cruikshank WW (2004). The effect of interleukin-16 and its precursor on T lymphocyte activation and growth. Growth Factor.

[60] McFadden C, Morgan R, Rahangdale S, Green D, Yamasaki H, Center D (2007). Preferential migration of T regulatory cells induced by IL-16. J Immunol.

[61] Zhang Y, Tuzova M, Xiao ZX, Cruikshank WW, Center DM (2008). Pro-IL-16 recruits histone deacetylase 3 to the Skp2 core promoter through interaction with transcription factor GABP. J Immunol.

[62] Richmond J, Tuzova M, Cruikshank W, Center D (2014). Regulation of cellular processes by interleukin-16 in homeostasis and cancer. J Cell Physiol.

[63] Center DM, Cruikshank WW, Parada NA, Ryan T, Theodore AC, Viglianti G (2001). Measurement of interleukin-16. Curr Protoc Immunol.

[64] Parada NA, Center DM, Kornfeld H, Rodriguez WL, Cook J, Vallen M (1998). Synergistic activation of CD4+ T cells by IL-16 and IL-2. J Immunol.

[65] Hermann E, Darcissac E, Idziorek T, Capron A, Bahr GM (1999). Recombinant interleukin-16 selectively modulates surface receptor expression and cytokine release in macrophages and dendritic cells. Immunology.

[66] Venet F, Chung CS, Huang X, Lomas-Neira J, Chen Y, Ayala A (2009). Lymphocytes in the development of lung inflammation: a role for regulatory CD4+ T cells in indirect pulmonary lung injury. J Immunol.

[67] Salamone G, Fraccaroli L, Gori S, Grasso E, Paparini D, Geffner J (2012). Trophoblast cells induce a tolerogenic profile in dendritic cells. Hum Reprod.

[68] Gu Y, Lewis DF, Deere K, Groome LJ, Wang Y (2008). Elevated maternal IL-16 levels, enhanced IL-16 expressions in endothelium and leukocytes, and increased IL-16 production by placental trophoblasts in women with preeclampsia. J Immunol.

[69] Giannubilo SR, Landi B, Pozzi V, Sartini D, Cecati M, Stortoni P (2012). The involvement of inflammatory cytokines in the pathogenesis of recurrent miscarriage. Cytokine.

[70] Benichou G, Tonsho M, Tocco G, Nadazdin O, Madsen JC (2012). Innate immunity and resistance to tolerogenesis in allotransplantation. Front Immunol.

[71] Beevers AJ, Dixon AM (2010). Helical membrane peptides to modulate cell function. Chem Soc Rev.

