# Supplementary material for: Cationic antimicrobial peptide, magainin down-regulates secretion of pro-inflammatory cytokines by early placental cytotrophoblasts
Source: Reprod Biol Endocrinol. 2015 Nov 6;13:121. doi: 10.1186/s12958-015-0119-8 (PMC4636767; doi:10.1186/s12958-015-0119-8)
Supplement: Additional file 1: Table S1. — Cytokines, chemokines and growth factors studied. (DOCX 18 kb) [file 12958_2015_119_MOESM1_ESM.docx]

Supplemental Table 1

Cytokines, chemokines and growth factors studied

___________________________________________________________________________

Name Full name Alternative name

___________________________________________________________________________

CCL2 Chemokine (C-C Motif) Monocyte Chemoattractant Protein 1

Ligand 2 (MCP1)

CCL3 Chemokine (C-C Motif) Macrophage Inflammatory Protein 1-alpha

Ligand 3 (MIP1A)

CCL4 Chemokine (C-C Motif) Macrophage Inflammatory Protein 1-beta

Ligand 4 (MIP1B)

CCL5 Chemokine (C-C Motif) Regulated Upon Activation, Normally T-

Ligand 5 Expressed, and Presumably Secreted

(RANTES)

CCL7 Chemokine (C-C Motif) Monocyte Chemoattractant Protein 3

Ligand 7 (MCP3)

CCL11 Chemokine (C-C Motif) Eosinophil Chemotactic Protein

Ligand 11 (Eotaxin-1)

CCL27 Chemokine (C-C Motif) Cutaneous T-cell-Attracting Chemokine

Ligand 27 (CTACK)

CXCL1 Chemokine (C-X-C Motif) Growth-Regulated alpha Protein

Ligand 1 (GROA)

CXCL9 Chemokine (C-X-C Motif) Monokine Induced by Interferon-Gamma

Ligand 9 (MIG)

CXCL10 Chemokine (C-X-C Motif) Interferon Gamma-Induced Protein

Ligand 10 (IP-10)

CXCL12 Chemokine (C-X-C Motif) Stromal Cell-Derived Factor 1

Ligand 12 (SDF-1)

FGF2 Fibroblast Growth Factor 2 Basic Fibroblast Growth Factor

(bFGF)

GCSF Granulocyte Colony-Stimulating Colony Stimulating Factor 3

Factor (CSF-3)

GMCSF Granulocyte-Macrophage Colony Colony Stimulating Factor 2

-Stimulating Factor (CSF-2)

HGF Hepatocyte Growth Factor Hepatocyte Growth Factor

(HGF)

IFNA2 Interferon, alpha 2 Interferon alpha-A (IFN-alphaA)

IFNG Interferon, gamma Immune Interferon (IFI)

IL1A Interleukin-1, alpha Hematopoietin-1

IL1B Interleukin 1, beta Catabolin

IL-1RA Interleukin 1 Receptor Antagonist Interleukin 1 Receptor Antagonist

(IL-1RN)

IL2 Interleukin 2 T-Cell Growth Factor (TCGF)

IL-2RA Interleukin 2 Receptor, alpha T-Cell Growth Factor receptor

(TCGFR)

IL3 Interleukin 3 Multipotential Colony-Stimulating Factor

(MULTI-CSF)

IL4 Interleukin 4 B-Cell Growth Factor 1 (BCGF1)

IL5 Interleukin 5 Eosinophil Differentiation Factor

(EDF)

IL6 Interleukin 6 Interferon beta-2 (IFNB2)

IL7 Interleukin 7 Interleukin 7

IL8 Interleukin 8 Chemokine (C-X-C Motif) Ligand 8

(CXCL8)

IL9 Interleukin 9 T-Cell Growth Factor p40 (HP40)

IL10 Interleukin 10 T-Cell Growth Inhibitory Factor (TGIF)

IL12p40 Interleukin 12 Subunit p40 Interleukin-12 subunit beta (IL12-beta)

IL12p70 Interleukin 12 (Holo) Natural Killer Cell Stimulatory Factor

(NKSF)

IL13 Interleukin 13 Interleukin 13

IL15 Interleukin 15 Interleukin 15

IL16 Interleukin 16 Lymphocyte Chemoattractant Factor

(LCF)

IL17 Interleukin 17 Cytotoxic T-Lymphocyte-Associated

Antigen 8 (CTLA8)

IL18 Interleukin 18 Interferon Gamma-Inducing Factor

(IGIF)

LIF Leukemia Inhibitory Factor Differentiation Inhibitory Activity

(DIA)

LTA Lymphotoxin alpha Tumor Necrosis Factor Beta

(TNF-beta)

MCSF Macrophage Colony-Stimulating Colony-Stimulating Factor 1 Factor (CSF1)

MIF Macrophage Migration Inhibitory Glycosylation-Inhibiting Factor

Factor (GIF)

NGFB Nerve Growth Factor, Beta Subunit beta-Nerve Growth Factor (bNGF)

PDGFB Platelet-Derived Growth Factor Platelet-Derived Growth Factor 2

Beta Polypeptide (PDGF2)

SCF Stem Cell Factor c-Kit ligand (c-KL)

SCGF Stem Cell Growth Factor Lymphocyte Secreted Long form

of C-type Lectin (LSLCL)

TNF Tumor Necrosis Factor Tumor Necrosis Factor-alpha

(TNF-alpha)

TRAIL TNF-Related Apoptosis Inducing Tumor Necrosis Factor (Ligand)

Ligand Superfamily, Member 10 (TNFSF10)

VEGF Vascular Endothelial Growth Vascular Endothelial Growth Factor A

Factor (VEGFA)

___________________________________________________________________________
