# Supplementary material for: Cationic antimicrobial peptide, magainin down-regulates secretion of pro-inflammatory cytokines by early placental cytotrophoblasts
Source: Reprod Biol Endocrinol. 2015 Nov 6;13:121. doi: 10.1186/s12958-015-0119-8 (PMC4636767; doi:10.1186/s12958-015-0119-8)
Supplement: Additional file 2: Table S2. — Characteristics of targets and primary antibodies used in the study. (DOCX 19 kb) [file 12958_2015_119_MOESM2_ESM.docx]

Supplemental Table 2

Characteristics of targets and primary antibodies used in the study

___________________________________________________________________________

Antigen Specification Final Purpose

(*Alias*) of antibody concentration

___________________________________________________________________________

Cytokeratin 7 Mouse IgG^a^ 6 μg/ml Epithelial cell marker, used in

(*CK-7*) ICC

CCL4 (*MIP1B*) Goat IgG^b^ 0.2 μg/ml Secretion affected by AMA, used

in WB

βhCG Rabbit IgG^c^ 10 μg/ml CTB differentiation marker, used

in ICC

TNF (*TNF alpha*) Mouse IgG^b^  0.4 μg/ml Secretion not affected by AMA,

used in WB

Vimentin (*Vim)* Mouse IgG^d^ 3 μg/ml Cytoskeletal protein marker for

fibroblasts, used in ICC

Vitronectin receptor, Mouse IgG^e^ 10 μg/ml Invasive CTB marker, used in (*CD51*) ICC

von Willebrand Rabbit IgG^c^ 25 μg/ml Endothelial cell marker (in

factor (*vWF*) Weibel-Palade bodies), used in

ICC

­­­­­­­­­­­­­­­­­­­­___________________________________________________________________________

CTB, cytotrophoblasts. ICC, immunocytochemistry. WB, Western blot analysis.

^a^Sigma-Aldrich, St Louis, MO, USA. ^b^Santa Cruz Biotechnology, TX, USA. ^c^Dako, Glostrup, Denmark. ^d^R&D Systems, Minneapolis. ^e^Zymed Laboratories Inc., San Francisco, CA, USA.
