# Supplementary material for: Cationic antimicrobial peptide, magainin down-regulates secretion of pro-inflammatory cytokines by early placental cytotrophoblasts
Source: Reprod Biol Endocrinol. 2015 Nov 6;13:121. doi: 10.1186/s12958-015-0119-8 (PMC4636767; doi:10.1186/s12958-015-0119-8)
Supplement: Additional file 3: Table S3. — List of genes and their primers used for quantitative real time RT-PCR. (DOC 34 kb) [file 12958_2015_119_MOESM3_ESM.doc]

Supplemental Table 3

List of genes and their primers used for quantitative real time RT-PCR

______________________________________________________________________

Gene symbol Accession number Primer sequences^1^

(mRNA RefSeq)

_______________________________________________________________________

CCL4 (MIP1B) NC_000017.11 TCCCCAATGCTCAATCACCC (s)

(NM_002984.3) GATCTCACCCTGGCCTTTCC (as)

CCL5 (RANTES) NC_000017.11 CCTCATTGCTACTGCCCTCT (s)

(NM_001278736.1) CGAACCCATTTCTTCTCTGG (as)

IL1A NC_000002.12 GCCTGCCTGTGCTTTTCTAC (s)

(NM_000575.3) TTGGTTGTTTGGGTTGTCTG (as)

IL1B NC_000002.12 CCCAGCCCTTTTGTTGAG (s)

([NM_000576.2](http://www.ncbi.nlm.nih.gov/nuccore/NM_000576.2)) CTACTTCTTGCCCCCTTTG (as)

IL-2RA NC_000010.11 CTCAGTACTTGCATCCTCTC (s)

([NM_000417.2](http://www.ncbi.nlm.nih.gov/nuccore/NM_000417.2)) AAATAGCTTTGTCCTCTGGG (as)

IL6 NC_000007.14 GTTTGTTTGGTTGGTTGG (s)

([NM_000600.3](http://www.ncbi.nlm.nih.gov/nuccore/NM_000600.3)) CTTGGTCTTTGTTTCTGTGG (as)

IL16  NC_000015.10 GCAGCTCGATTCTCTTCTAA (s)

([NM_001172128.1](http://www.ncbi.nlm.nih.gov/nuccore/NM_001172128.1)) TCCAAGCCTCTACTTTTGTC (as)

GAPDH NM_002046 ACAGTCAGCCGCATCTTC (s)

([NM_001256799.2](http://www.ncbi.nlm.nih.gov/nuccore/NM_001256799.2)) CTCCGACCTTCACCTTCC (as)

MIF NC_000022.11 TATTACGACATGAACGCGGC (s)

([NM_002415.1](http://www.ncbi.nlm.nih.gov/nuccore/NM_002415.1)) ACCGTTTATTTCTCCCCACCA (as)

TNF NC_000006.12 ATCTTGGGTAAAATGGTGTCC (s)

([NM_000594.3](http://www.ncbi.nlm.nih.gov/nuccore/NM_000594.3)) GTGTACTCCCTGGTCACTC (as)

UBC  M26880 CCTGGTGCTCCGTCTTAG (s)

([NM_021009.6](http://www.ncbi.nlm.nih.gov/nuccore/NM_021009.6)) GATGCCTTCCTTGTCTTGG (as)

___________________________________________________________________________

^1^Primers were designed using Beacon Designer Software (Premier Biosoft, Palo Alto, CA, USA) based on gene sequences corresponding to the genes, retrieved from NCBI data base. as, anti-sense. s, sense.
